# Frailty Prevalence and Related Factors among Older Adults in the Republic of Korea and the Need for Tailored Community-based Frailty Prevention Strategies

**DOI:** 10.56786/PHWR.2026.19.41suppl.1

**Published:** 2026-10-29

**Authors:** Insob Hwang, Kwangsuk Park, Ki-Soo Park, Youmi Kim

**Affiliations:** 1 Division of Chronic Disease Control, Department of Chronic Disease Prevention and Control, Korea Disease Control and Prevention Agency Cheongju Korea; 2 Department of Preventive Medicine, College of Medicine, Gyeongsang National University Jinju Korea

**Keywords:** Frailty, Sarcopenia, Aged, Frailty prevention, Community health services

## Abstract

**Objectives**: This study proposes tailored, local government-centered frailty prevention strategies that emphasize early frailty management in a super-aged society and consider sociodemographic characteristics, as well as domestic and international cases.

**Methods**: National statistics, including the National Survey of Older Koreans and the National Health and Nutrition Examination Survey, as well as domestic and international policy and project reports, were comprehensively reviewed to assess frailty prevalence and current public health center programs.

**Results**: Frailty and pre-frailty prevalence among older adults aged ≥65 were 4.6% and 32.2%, respectively, with clear differences across sociodemographic characteristics such as sex, age, residential environment, and household type. Frailty shows “reversible dynamics” responsive to intervention, and preventive programs have demonstrated effectiveness in international settings and in some domestic local governments in the Republic of Korea. However, current public health center programs focus mainly on vulnerable groups, limiting early risk detection because they lack standardized manuals and regional, data-based response systems.

**Conclusions**: Based on national institutional support from the Korea Disease Control and Prevention Agency, including frailty surveys and standardized manuals, local governments should strengthen their capacity to implement guidelines and deliver region-specific programs. In addition, an effective frailty prevention framework requires a supportive community environment, public-private collaboration among resident volunteers and welfare/medical systems, and a sustainable follow-up management system.

**Key messages**

**① What is known previously?**

Frailty (prevalence 4.6%; pre-frailty 32.2%) is reversible with early intervention; however, existing public health center programs primarily target vulnerable groups, limiting early identification of at-risk individuals.

**② What new information is presented?**

Frailty risk varies by sociodemographic characteristics, including sex, age, region of residence, and household type. Furthermore, a national institutional framework is being established to help local governments implement evidence-based frailty prevention programs, including the Korea Disease Control and Prevention Agency’s dissemination of standardized manuals and the production of regional frailty statistics.

**③ What are implications?**

Local governments should strengthen their capacity to flexibly adapt and plan national guidelines to regional needs and establish sustainable follow-up management systems through public-private partnerships.

## Introduction

As the Republic of Korea (ROK) enters a super-aged society at an unprecedented rate globally, with the population aged 65 and older exceeding 20% of the total population in 2024 [1], public health policies for older adults are also facing a fundamental paradigm shift. While the focus was previously on the prevention and management of individual chronic diseases, such as hypertension and diabetes, among older adults, the prevention and early management of “frailty,” characterized by the combined decline of physical, mental, and social functioning in old age, is emerging as an important public health challenge in a super-aged society [2,3]. Frailty is a state of reduced physiological resilience to external stressors that, if left unaddressed, substantially increases the risk of requiring care, hospitalization, or death. However, frailty is not an irreversible aging process but has the potential for reversal, allowing individuals to return to a normal state if appropriate interventions are provided during the pre-frailty and early frailty stages [4].

Regarding frailty prevalence in the ROK and other countries, the 2023 National Survey of Older Koreans reported a frailty prevalence of 4.6% and a pre-frailty rate of 32.2% among adults aged 65 and older, indicating that more than one-third of older adults fall into the frailty or pre-frailty risk group [5]. In other countries, frailty is also emerging as a major public health challenge because of rapid population aging. In Singapore, the prevalence of frailty among adults aged 60 and older is 6.2%, and the pre-frailty rate is 37.0% [6]. Japan, which entered a super-aged society before the ROK, has a higher reported frailty prevalence of 8.7% and a pre-frailty rate of 40.8% [7]. However, direct comparisons of these figures are limited by differences in measurement tools and assessment criteria across countries.

Meanwhile, the onset and progression of frailty may vary according to sociodemographic characteristics, such as sex, age, residential area, and household structure. Accordingly, this study aims to examine the associations between frailty prevalence, related factors, and the sociodemographic characteristics of older adults in the ROK; review major domestic and international policy implementation cases; and highlight the need to promote community-tailored frailty prevention programs and develop effective policy measures.

## Methods

This study used nationally approved statistics, press releases, domestic and international policy reports, academic papers, program outcome reports, and white papers to identify the prevalence of frailty and sarcopenia according to sociodemographic characteristics and propose program directions based on domestic and international policy and program experiences.

To examine frailty characteristics and related health indicators among older adults in the ROK, two sets of nationally approved statistical data with different purposes and measurement methods were used complementarily. The Ministry of Health and Welfare’s 2023 National Survey of Older Koreans provides information on frailty screening status (e.g., using the K-FRAIL scale) among the older adult population nationwide, whereas the Korea National Health and Nutrition Examination Survey (KNHANES), conducted by the Korea Disease Control and Prevention Agency (KDCA), provides indicators of sarcopenia (e.g., low handgrip strength), physical activity, and oral health based on physical measurements and functional tests. Based on these data, key indicators were classified, compared, and summarized, including frailty prevalence by residential area, sex, age group, and household structure among adults aged 65 and older, as well as sarcopenia prevalence, the prevalence of low handgrip strength, the walking practice rate, the prevalence of depressive disorders, and the proportion of older adults reporting chewing discomfort. However, because the two surveys differ in their target populations and measurement tools, the results were interpreted in consideration of the definitions and limitations of each indicator.

Next, trends in domestic and international frailty management policies and programs were reviewed. Internationally, guidelines from the World Health Organization (WHO) and examples from Japan and Singapore, which were among the early adopters of frailty prevention programs in Asia, were examined to identify community-based frailty screening and management systems. In addition, the United Kingdom’s primary care-based approach to frailty prevention and management was described. Domestically, because no nationally led frailty prevention program was in place, research papers, white papers, program outcome reports, academic symposium presentations, and commissioned policy research reports concerning frailty prevention programs implemented by local governments in Pyeongchang-gun, Gangwon-do; Busan Metropolitan City; and Namhae-gun, Gyeongsangnam-do, were reviewed.

In addition, a commissioned policy research report funded by the KDCA and conducted by the Industry-Academic Cooperation Foundation of Gyeongsang National University was reviewed to identify and describe the structural limitations faced by practitioners directly responsible for older adult health services at local public health institutions, such as public health centers, and the need to establish a standardized manual.

## Results

### 1. Frailty Prevalence Status and Related Factors among Older Adults in the Republic of Korea

#### 1) Age-specific characteristics by sex

As of 2023, the prevalence of frailty among adults aged 65 and older in the ROK was 4.6% (2.8% among males and 5.9% among females), whereas the prevalence of sarcopenia, which is known to contribute significantly to physical frailty, was 9.4% as of 2024 (9.5% among males and 9.3% among females) [5,8]. Frailty prevalence and related physical indicators, such as handgrip strength, show contrasting patterns by sex. Among adults in the early stage of old age (aged 65–69 years), the prevalence of sarcopenia (5.8%) is higher among females than among males (2.6%), suggesting that physical health risks emerge at a relatively younger age among females [9]. This may be attributable to a combination of factors, including lower baseline muscle mass, muscle loss following the decline in estrogen levels after menopause, a higher prevalence of chronic diseases, and relatively disadvantaged socioeconomic conditions, which may expose females to the risk of muscle strength loss at an earlier age [10]. Conversely, males tend to maintain relatively favorable health status during early old age but exhibit a “sex reversal phenomenon,” in which sarcopenia prevalence rises sharply after age 70 and exceeds that among females [9]. However, among adults aged 80 and older, the prevalence of low handgrip strength remains higher among females (41.5%) than among males (36.8%), indicating that females’ vulnerability in muscle strength persists into the later stages of life [9]. Taken together, females tend to develop frailty at a relatively younger age, whereas males exhibit a different pattern, with prevalence increasing in later life in association with rapid muscle loss and the accumulation of chronic diseases.

#### 2) Differences in related health issues by age group

According to data published by the KDCA [9], an analysis of the health risks associated with low handgrip strength, stratified by age group into the “younger-old” (65–74 years) and “older-old” (75 years and older), revealed different patterns of health risks across the two groups. Among adults in the younger-old group (65–74 years), the prevalence of depressive disorders was 14.3% in the low handgrip strength group, more than seven times the prevalence in the normal handgrip strength group (2.0%). This suggests a strong association between declining muscle strength in early old age and mental health issues, particularly depression, potentially in conjunction with retirement, loss of social roles, and feelings of social isolation, rather than physical functional decline alone. In contrast, among adults in the older-old group (75 years and older), low handgrip strength was associated with reduced physical activity and imbalances in nutritional intake. The proportion of individuals who did not engage in walking practice (64.4%) and the proportion reporting chewing discomfort (42.0%) in the low handgrip strength group were higher than those in the normal handgrip strength group (54.1% and 32.6%, respectively). In other words, in later life, muscle weakness may contribute to a cycle of reduced physical activity, impaired oral function, inadequate protein intake, and worsening sarcopenia (Table 1).

#### 3) Differences between urban and rural areas

The National Survey of Older Koreans [5] also identified residential area and household type as important factors associated with frailty prevalence. An analysis by residential area revealed that the prevalence of frailty (6.0%) and pre-frailty (36.1%) among older adults in rural areas (eup and myeon regions) was higher than that in urban areas (dong regions), where the corresponding prevalence rates were 4.1% for frailty and 30.9% for pre-frailty. This may be attributable to environmental factors, including higher population aging rates in rural areas, limited access to health care and exercise facilities, and social isolation, which may contribute to frailty progression. In addition, frailty prevalence among older adults living alone was 7.1%, more than 2.5 times the rate among older adults living with a spouse (2.8%). Living alone may contribute to nutritional imbalances because of inadequate meals, reduced physical activity, and social isolation, potentially leading to depression and cognitive decline. Therefore, future preventive policies should carefully consider vulnerabilities associated with household characteristics rather than relying solely on age-based criteria (Table 1).

### 2. Overseas Frailty Management Policy Trends and Domestic Local Government Frailty Prevention Project Cases

#### 1) Overseas policy trends: WHO guidelines and primary care/community-centered systems

In response to the changing needs associated with population aging, the WHO recommended shifting the focus from disease treatment alone toward preserving “intrinsic capacity,” which encompasses physical and mental functioning in older age, and promoting “healthy ageing.” In 2017, the WHO developed and disseminated the Integrated Care for Older People guidelines to support the early screening and management of frailty and functional decline in primary care and community settings [3]. Building on this approach, several countries have been actively establishing community-based frailty management systems. In Singapore, a national frailty response strategy links early screening in primary care clinics by general practitioners with integrated exercise, nutrition, and social networking programs at community-based Active Ageing Centres [11]. Japan has established a public–private collaboration framework that leverages community resources, in which resident volunteers known as “frailty supporters (フレイルサポーター)” conduct local frailty assessments and connect residents with health and welfare services [12]. The United Kingdom promotes primary care- and community-based frailty prevention programs by incorporating early screening using the electronic frailty index and the development of individualized care plans into primary care physicians’ contracts [13].

#### 2) Domestic local government cases: preemptive project implementation and achievements

In the ROK, the development of a unified national frailty prevention policy and standardized program framework remains at an early stage. However, some local governments with high population aging rates have implemented proactive frailty prevention programs, demonstrating promising results. In Pyeongchang-gun, Gangwon-do, a 24-week, multidomain frailty prevention intervention implemented through collaboration between a public health medical center and university hospital researchers was reported to extend the “disability-free survival period” of participating older adults by 6.5 months and reduce medical and long-term care costs by approximately KRW 10 million per person, with a cost-benefit ratio of approximately 8.8:1 [14,15]. As part of the KDCA’s “Small-Area Health Disparity Relief Project,” Namhae-gun, Gyeongsangnam-do, provided integrated frailty prevention exercise classes, protein supplementation, and home-visit health care services to residents of myeon-level areas characterized by poor health outcomes and a high burden of chronic disease, thereby improving participants’ physical function and nutritional status [16]. In addition, Busan Metropolitan City actively operates a community-based frailty prevention model that leverages dong-level village health centers and community health partners [17].

### 3. Limitations of Community-centered Frailty Prevention Programs

Although the potential for recovery from frailty and the effectiveness of multidomain community-based interventions have been demonstrated through programs implemented by various local governments, many public health centers face the following structural challenges in implementing frailty prevention programs.

#### 1) Limitations of proactive identification of frailty and preventive interventions

One fundamental concern raised by various health care professionals is that the current community health system focuses primarily on reactive responses centered on treatment and care. Currently, frailty management at public health centers is fragmented, focusing primarily on home-visit health care services for vulnerable populations and general health promotion programs. Pathways for the early identification of individuals with initial functional decline and their referral to preventive programs are limited [18-20]. Furthermore, personnel responsible for home-visit health care services at public health centers are already heavily burdened by the management of vulnerable populations, limiting their capacity to provide proactive interventions to all newly identified candidates for frailty prevention programs [21]. As a result, although the primary goal of frailty management is to promote functional recovery through timely interventions during the pre-frail and early frailty stages, a recurring limitation in practice is that management often begins only after health vulnerabilities have worsened or frailty has progressed to a severe stage.

#### 2) Lack of standardized manuals and limitations on project sustainability

The second limitation is the lack of standardized program manuals for field practitioners, together with limitations in project managers’ planning and operational capacities. According to a survey of public health center program managers, a “lack of program planning and operational capacity” was cited as the greatest difficulty in implementing frailty prevention programs, and 25.4% of respondents identified the “provision of standardized program guidelines” as their highest-priority support need [22]. Furthermore, some successful initiatives implemented by local governments have relied heavily on the commitment of local government leaders and public health center directors, the individual capabilities of program managers, or close support from external experts, such as university researchers. Consequently, these projects faced challenges in maintaining continuity when personnel were transferred or external support ended. This highlights the urgent need for national-level standardized manuals that include screening and assessment tools, evidence-based intervention programs, and performance evaluation guidelines that field practitioners can readily implement.

#### 3) Absence of local-level frailty data for evidence-based planning

Finally, the lack of local government-level data on frailty status presents a barrier to evidence-based planning. Historically, information on frailty prevalence has been derived primarily from national surveys, such as the National Survey of Older Koreans and the KNHANES, as well as findings from individual studies. Consequently, public health centers lack local government-level statistics that allow frailty and pre-frailty prevalence to be analyzed in conjunction with related sociodemographic factors. This lack of data creates fundamental difficulties in objectively assessing local frailty conditions and planning evidence-based, customized programs. Because frailty prevalence patterns vary according to sex, age, living environment, and household type, developing tailored strategies based on local data is essential to maximizing program effectiveness.

## Conclusion

Frailty has the potential for reversal, allowing individuals to return to a normal state if it is detected early and appropriate interventions are provided during the pre-frailty and early frailty stages. However, public health centers currently focus heavily on general health management services, such as home-visit health care services targeting vulnerable populations and integrated health promotion programs, which limits their ability to extend services to the broader older adult population and groups at an early stage of risk. In addition, they face difficulties in establishing a proactive frailty prevention system because of the lack of standardized frailty program manuals and region-specific data. As confirmed by the survey of public health center personnel, the identification of “providing standardized program guidelines” as the top priority clearly underscores the urgent need to establish standardized guidelines that public health centers nationwide can readily implement.

Fortunately, efforts to establish a policy framework to address these practical limitations have recently gained momentum under the leadership of the KDCA. To address the previous lack of a unified national standard for frailty prevention programs in the ROK, the KDCA is developing the “National Frailty Definition Standard” and “Frailty Awareness Improvement Messages” to raise public awareness of frailty and is leading efforts to prepare a “standard program manual” that public health centers can readily implement [23]. In addition, the introduction of the Korean frailty screening tool (K-FRAIL) indicator into the Community Health Survey in 2026 established an institutional foundation for generating objective statistics on frailty status at the local government level nationwide [24].

In light of this policy development, local governments should carefully analyze the characteristics of their older adult populations by sex, age, residential area, and household type using future Community Health Survey data and actively incorporate these findings into program planning. Because frailty risk levels and appropriate intervention strategies vary according to sociodemographic characteristics, the central government should provide standardized guidelines to ensure minimum service quality while strengthening the planning and operational capacities of local governments so that each can independently set priorities and develop tailored programs based on its demographic profile and regional characteristics.

In addition, effective frailty prevention requires fostering a community-wide understanding that frailty is not an inevitable consequence of aging but a condition that can be reversed through prevention and appropriate management. Proactive identification of at-risk individuals and voluntary resident participation can be encouraged by actively disseminating frailty self-assessment tools and prevention guidelines through settings that older adults regularly visit, such as senior centers, welfare centers, and village halls, while fostering a health-promoting environment in which community members take responsibility for their own health.

Furthermore, because the limited number of dedicated personnel at public health centers cannot directly manage the rapidly expanding population at risk of frailty, community human and material resources—including resident volunteers, personnel involved in older adult employment programs, local health care and medical institutions, and welfare facilities—should be coordinated and mobilized. Drawing on international examples, such as Japan’s “frailty supporters,” and local initiatives, such as Busan’s “community health partners,” these efforts should facilitate the development of a functional public–private partnership ecosystem in which the entire community participates in frailty prevention.

In particular, as demonstrated by domestic examples, establishing a sustainable follow-up management system, such as peer support groups, is important to ensure that improvements in physical function and health behaviors are maintained after intervention programs end. The long-term effects of interventions should be sustained by connecting program completers with local peer support groups, senior center clubs, and community exercise classes and establishing a continuous support system in which resident leaders or health partners facilitate ongoing exercise and nutritional management.

Ultimately, a sustainable, region-specific frailty prevention system capable of addressing the challenges of a super-aged society can be established only by combining a national foundation of standardized manuals and public awareness initiatives with local governments’ capacity to develop data-driven, tailored programs and a public–private collaboration system that mobilizes community resources.

## Figures and Tables

**Table 1:** Main status and risk factors related to frailty in older adults

Category		Main status	Possible explanations
Sex	Female	- Frailty at 5.9%a) among aged ≥65 years, sarcopenia at 5.8%b) among 65–69 years (more than twice that of males) - Low handgrip strength rate 41.5% among adults aged ≥80 yearsb)	Early onset and long-term persistence into old age, driven by low baseline muscle mass, hormonal changes, and socioeconomic vulnerability
	Male	- Frailty at 2.8%a) among aged ≥65 years, sarcopenia at 2.6%b) among 65–69 years - Rapid increase in sarcopenia risk after age 70b)	A steep increase in frailty risk after age 70 due to rapid muscle loss and the accumulation of chronic diseases
Age group	Young-old (ages 65–74)	- Prevalence of depressive disorder in the low handgrip group: 14.3%b) (more than seven times higher than the normal group at 2.0%)	Loss of physical strength and social roles (such as retirement) are closely associated with increased risk of mental health issues (depression)
	Old-old (ages 75 and over)	- Non-walking practice rate in the low handgrip group: 64.4%b) (vs. 54.1% in the normal group) - Chewing discomfort rate: 42.0%b) (vs. 32.6% in the normal group)	A vicious cycle of physical and nutritional decline: involving reduced physical activity+impaired oral function+poor nutrition
Environmental and household characteristics	Rural areas	- Frailty 6.0%, pre-frailty 36.1%a) (vs. urban areas: 4.1%/30.9%)	Advanced age, limited access to healthcare and exercise infrastructure, and social isolation
	Single-person households	- Frailty 7.1%a) (>2.5 times higher than elderly couple households at 2.8%)	Poor nutritional intake, emotional loneliness, and reduced physical activity accelerate depression and cognitive decline

^a)^Data from Kang et al. (2023 National Survey of Older Koreans; 2023) [5]. ^b)^Data from the Korea Disease Control and Prevention Agency (https://www.kdca.go.kr/bbs/kdca/42/312118/artclView.do) [9].
