# Supplementary material for: Frailty Prevalence and Related Factors among Older Adults in the Republic of Korea and the Need for Tailored Community-based Frailty Prevention Strategies
Source: Public Health Wkly Rep. 2026 Oct 29;19(41 Suppl):1–18. doi: 10.56786/PHWR.2026.19.41suppl.1 (PMC13724135; doi:10.56786/PHWR.2026.19.41suppl.1)
Supplement: Translation

# Translation: 우리나라 노인의 노쇠 현황 및 관련 요인과 지역사회 맞춤형 노쇠 예방 전략의 필요성

**Authors:** 인섭 황, 광숙 박, 기수 박, 유미 김

**Affiliations:** 1 질병관리청 만성질환관리국 만성질환관리과; 2 경상국립대학교 의과대학 예방의학교실

**Keywords:** 노쇠, 근감소증, 노인, 노쇠 예방, 지역 보건 서비스

## Abstract

**목적:**

초고령사회 진입에 따른 ‘노쇠(frailty)’의 선제적 예방과 조기 관리 중요성을 바탕으로 인구사회학적 특성에 따른 유병 특성을 확인하고 국내외 사례를 통해 지자체 중심의 맞춤형 노쇠 예방 전략 마련의 필요성을 제안하고자 한다.

**방법:**

노인실태조사, 국민건강영양조사 등 국가 승인 통계와 국내외 정책‧사업 결과 보고서를 검토하여, 인구사회학적 특성별 노쇠 유병 현황과 보건소 사업 현황을 검토하였다.

**결과:**

65세 이상 노인의 노쇠 유병률은 4.6%, 전노쇠는 32.2%이며 성별, 연령별, 거주 지역 환경, 가구 구성 등 인구사회학적 특성에 따라 유병 양상의 차이를 보인다. 노쇠는 초기 개입 시 회복 가능한 ‘가역적 역동성’을 지니며, 국외 및 국내 일부 지자체에서 사업의 효과를 확인한 바 있다. 다만, 현행 보건소 사업은 취약계층 중심 관리에 치중되어 초기 위험군 발굴에 한계가 있으며, 표준 매뉴얼과 지역 데이터 기반 대응체계의 부재로 현장의 어려움이 예상된다.

**결론:**

질병관리청의 노쇠 현황 조사와 보건소 표준 매뉴얼 보급 등 국가 차원의 제도적 기반을 바탕으로, 지자체는 표준 가이드라인을 지역 실정에 맞게 적용하고 사업을 기획‧운영할 수 있는 역량을 강화해야 한다. 아울러 노쇠 예방이 일상 속에서 이루어지는 분위기를 조성하고, 주민 봉사자 및 복지‧의료 인프라를 활용한 민관 협력을 통해 단기 중재 후에도 작동하는 ‘지속 가능한 사후관리 체계’를 구축함으로써 실효성 있는 노쇠 예방 전략을 완성해야 할 것이다.

**핵심요약**

**① 이전에 알려진 내용은?**

노쇠(유병률 4.6%, 전노쇠 32.2%)는 초기 개입 시 회복 가능하나, 기존 보건소 사업은 취약계층 중심 관리에 치중되어 초기 위험군 발굴에 한계가 있었다.

**② 새로이 알게 된 내용은?**

성별, 연령, 거주지, 가구 형태 등 인구사회학적 특성에 따라 노쇠 유병 양상이 다르며, 최근 질병관리청의 표준 매뉴얼 보급, 지역별 노쇠 현황 산출 등 지자체의 근거 중심 노쇠 예방 사업 추진을 지원하는 국가 차원의 제도적 기반이 마련되고 있다.

**③ 시사점은?**

지자체는 국가 표준 가이드라인을 지역 실정에 맞게 유연하게 적용·기획하는 역량을 강화하고, 민관 협력 기반의 지속 가능한 사후관리 체계 구축이 필요할 것이다.

## 서 론

우리나라는 2024년 65세 이상 노인 인구가 전체의 20%를 넘어서며 전 세계적으로 유례없이 빠르게 초고령사회에 진입함에 따라[1], 노인 대상 공중보건 정책 역시 근본적인 패러다임 전환의 시점을 맞이하고 있다. 기존에는 노인들을 대상으로 고혈압, 당뇨병 등 단일 만성질환의 예방과 관리에 중점을 두었다면, 초고령사회에서는 노년기 전반의 신체적‧정신적‧사회적 기능 저하가 복합적으로 나타나는 ‘노쇠(frailty)’의 예방과 조기 관리가 중요한 보건 과제로 부상하고 있다[2,3]. 노쇠는 외부 스트레스에 대한 생리적 회복력이 저하된 상태로, 방치할 경우 돌봄 필요 상태나 입원, 사망의 위험을 급격히 높인다. 그러나, 노쇠는 불가역적인 노화 과정이 아니라 전노쇠(pre-frailty) 및 조기 노쇠 단계에서 적절한 중재를 제공할 경우 정상 상태로 회복 가능한 ‘가역적 역동성’을 지닌다[4].

국내외 노쇠 유병 현황을 살펴보면 국내의 경우 2023년 노인실태조사 결과 65세 이상 노인의 노쇠 유병률 4.6%, 전노쇠 비율은 32.2%로 노인 3명 중 1명 이상이 노쇠 또는 노쇠 위험군에 해당하는 것으로 나타났다[5]. 해외 주요국 역시 급격한 고령화에 따라 노쇠 문제가 주요한 보건 과제로 대두되고 있다. 싱가포르의 경우 60세 이상 노인의 노쇠 유병률이 6.2%, 전노쇠 비율이 37.0%에 달하며[6], 우리나라보다 먼저 초고령사회에 진입한 일본은 노쇠 유병률 8.7%, 전노쇠 40.8%로 한층 높은 수준을 보이고 있으나[7], 이는 국가별 측정 도구 및 평가 기준의 차이가 존재하므로 수치의 직접 비교에는 한계가 있다.

한편, 노쇠의 발생과 진행 양상은 성별, 연령, 거주 지역 유형, 가구 형태 등 인구사회학적 특성에 따라 차이를 보일 수 있다. 이에 본 원고에서는 국내 노인의 인구사회학적 특성에 따른 노쇠의 유병 특성 등 관련 요인과의 연관성을 확인하고, 국내외 주요 정책 추진 사례를 검토함으로써 향후 지역사회 맞춤형 노쇠 예방 사업 추진의 필요성과 실효성 있는 정책적 고려 사항을 제안하고자 한다.

## 방 법

본 원고는 인구사회학적 특성에 따른 노쇠 및 근감소증의 유병 특성을 파악하고 국내외 정책 및 사업 경험을 바탕으로 사업 방향을 제안하고자 국가 승인 통계, 보도자료, 국내외 정책 보고서, 학술 논문 및 사업 결과 보고서, 백서 등을 종합적으로 활용하였다.

국내 노인의 노쇠 및 관련 건강 지표의 특성을 검토하기 위해서는 목적과 측정 방식이 다른 두 가지 국가 승인 통계 자료를 상호 보완적으로 사용하였다. 보건복지부의 「2023년 노인실태조사」는 전국 노인을 대상으로 노쇠 선별(K-FRAIL 등) 현황을 파악하고 있으며, 질병관리청의 「국민건강영양조사」는 신체 계측 및 기능 검사를 기반으로 측정된 근감소증(악력 저하 등), 신체활동, 구강건강 상태 등 지표를 산출하고 있다. 이를 바탕으로 65세 이상 노인의 거주지, 성별, 연령대별, 가구 형태에 따른 노쇠 유병률과 65세 이상 고령층의 근감소증 유병률과 악력 저하율, 걷기 실천율, 우울장애 유병률, 저작 불편 호소율 등의 주요 지표를 구분하여 비교‧정리하였다. 하지만 두 조사는 대상 범위 및 측정 도구에 차이가 있으므로, 각 지표의 정의와 한계를 고려하여 결과를 해석하였다.

이어 국내외 노쇠 관리 정책 동향과 사업 현황을 검토하였다. 국외의 경우 세계보건기구(World Health Organization, WHO)의 지침과 아시아 국가 중 노쇠 예방 사업을 먼저 시작한 일본, 싱가포르 사례를 검토하여 지역사회 중심의 노쇠 스크리닝 및 관리 체계를 확인하고, 영국의 사례를 통해 일차의료 중심 노쇠 예방 관리 체계에 대해서도 기술하였다. 국내의 경우 국가 주도의 노쇠 예방 사업이 없어, 지자체 주도 노쇠 예방 사업 추진 사례로 많이 알려진 강원 평창군, 부산광역시, 경남 남해군의 연구 논문, 백서, 사업 결과 보고서, 학술 심포지엄 발표자료, 정책연구용역 보고서 등을 검토하였다.

또한, 질병관리청의 지원으로 경상국립대학교 산학협력단에서 수행한 정책연구용역 보고서를 통해 보건소 등 지역 보건의료기관에서 노인 건강 관련 업무를 직접 담당하는 현장 실무자가 직면한 구조적 한계점과 표준 매뉴얼 구축의 필요성을 확인하여 제시하였다.

## 결 과

### 1. 우리나라 노인의 노쇠 유병 현황과 관련 요인

#### 1) 성별에 따른 연령별 특성

우리나라 65세 이상 노인의 노쇠 유병률은 2023년도 기준 4.6% (남자 2.8%, 여자 5.9%)이며, 신체적 노쇠에 영향을 크게 미치는 것으로 알려진 근감소증 유병률은 2024년도 기준 9.4% (남자 9.5%, 여자 9.3%)이다[5,8]. 노쇠 유병 및 관련 신체 지표(악력 등)는 성별에 따라 대조적인 양상을 보인다. 노인 연령 초반인 65–69세에서는 여성의 근감소증 유병률(5.8%)이 남성(2.6%)보다 높게 관찰되어, 상대적으로 이른 연령대부터 신체적 위험이 나타나는 경향을 보인다[9]. 이는 여성의 경우 낮은 기초 근육량, 폐경 후 에스트로겐 감소에 따른 근 손실, 높은 만성질환 유병률 및 상대적으로 취약한 사회경제적 여건 등이 복합적으로 작용하여 근력 저하 위험에 일찍 노출되기 때문이다[10]. 반면, 남성은 전기 고령까지 상대적으로 양호한 건강 상태를 유지하다가 70세 이후 근감소 유병률이 급증하면서 여성을 추월하는 역전 현상을 보인다[9]. 다만, 80세 이상 초고령에서는 여성의 악력 저하율(41.5%)이 남성(36.8%)을 계속 상회하여, 여성의 근력 취약성이 생애 후반에 걸쳐 지속됨을 알 수 있다[9]. 이를 종합하면, 여성은 상대적으로 이른 시기에 초기 노쇠로 진입하는 반면, 남성은 후기 고령기 이후 급격한 근 손실과 만성질환 누적으로 상대적으로 높은 유병률을 나타내는 차이를 보인다.

#### 2) 연령군에 따른 관련 건강 문제의 차이

질병관리청에서 발표한 자료[9]에 따르면 고령화의 단계를 ‘전기(65–74세)’와 ‘후기(75세 이상)’로 구분하여 악력 저하에 따른 복합 건강 위험을 분석한 결과, 연령대에 따라 관찰되는 위기 양상이 다르게 나타났다. 전기 고령자(65–74세) 중 악력 저하군의 우울장애 유병률은 14.3%로, 정상 악력군(2.0%) 대비 7배 이상 높았다. 이는 전기 고령기의 근력 저하가 단순한 신체기능 하락에 그치지 않고, 은퇴 및 사회적 역할 상실, 사회적 고립감과 맞물려 정신건강(우울) 문제와의 높은 관련성을 시사한다. 반면, 후기 고령자(75세 이상)에서는 악력 저하가 신체활동 감소 및 영양 섭취 불균형과 연계되는 양상을 보였다. 악력 저하군의 걷기 운동 미실천율(64.4%)과 저작 불편 호소율(42.0%)은 정상군(각각 54.1%, 32.6%)을 크게 상회하였다. 즉, 후기 고령기에서는 근력 약화가 신체활동 감소, 구강 기능 저하, 단백질 섭취 부족 및 근감소증 악화로 이어지는 구조를 형성하고 있음을 예상해 볼 수 있다(표 1).

#### 3) 도시와 농촌 간 차이

노인실태조사[5]에서는 거주 지역과 가구 형태 역시 노쇠 유병률을 결정하는 중요한 요인으로 확인된다. 거주 지역별 분석 결과, 농어촌(읍‧면 지역) 노인의 노쇠 유병률(6.0%)과 전노쇠 비율(36.1%)은 도시(동 지역, 노쇠 4.1%, 전노쇠 30.9%)에 비해 높게 나타났다. 이는 농어촌 지역 노인의 높은 고령화율과 더불어 보건의료 및 운동 인프라의 접근성 한계, 사회적 교류 단절 등이 노쇠를 가속화하는 환경적 요인으로 작용했기 때문이다. 또한 독거노인의 노쇠 유병률은 7.1%로 노인부부 가구(2.8%) 대비 2.5배 이상 높은 수준을 보였다. 홀로 거주하는 환경은 부실한 식사로 인한 영양 불균형, 신체활동 감소, 정서적 고립을 유발하여 우울 및 인지 기능 저하로 이어지므로, 향후 예방 정책 추진 시 단순 연령 기준을 넘어 가구 특성별 취약성을 면밀히 고려해야 한다(표 1).

### 2. 국외 노쇠 관리 정책 동향과 국내 지자체 노쇠 예방 사업 사례

#### 1) 국외 정책 동향: WHO 지침 및 일차의료‧지역사회 중심 체계

WHO는 고령화 대응 패러다임의 변화에 따라 단순 질환 치료 중심에서 벗어나 노년기 신체적‧정신적 기능 상태인 ‘내재적 역량(intrinsic capacity)’을 보존하고 ‘건강 노화(healthy ageing)’를 도모할 것을 권고하며, 일차의료 및 지역사회 현장에서 노쇠와 기능 저하를 조기에 선별‧관리할 수 있도록 2017년에 노인 통합관리지침(Integrated Care for Older People)을 개발하여 보급하였다[3]. 이를 기반으로 주요국들은 지역사회 중심의 노쇠 관리 체계를 활발히 구축하고 있다. 싱가포르에서는 국가 노쇠 대응 전략을 통해 일차의료기관에서 일반의(general practitioner)가 시행하는 조기 스크리닝과 지역사회 Active Ageing Centre의 운동‧영양‧사회관계망 통합 프로그램을 연계하여 운영 중이다[11]. 일본은 주민 봉사자인 ‘프레일 서포터(フレイルサポーター)’가 지역 내 프레일 체크를 수행하고, 이를 보건‧복지 인프라와 연결하는 등 지역사회 자원을 활용한 민관협력 생태계를 갖추고 있으며[12], 영국은 일차의료 의사 계약 시 전자 노쇠 지수(electronic frailty index)를 활용한 조기 선별과 맞춤형 케어플랜 작성을 의무화하여 지역사회 일차의료 중심의 노쇠 예방 사업을 추진하고 있다[13].

#### 2) 국내 지자체 사례: 선제적 사업 추진 및 성과

국내의 경우 국가 차원의 통일된 노쇠 예방 정책 및 표준 사업 체계 구축은 초기 단계이나, 고령화율이 높은 일부 지자체를 중심으로 선제적인 노쇠 예방 사업이 추진되어 긍정적인 가능성을 제시해 왔다. 강원 평창군은 보건의료원과 대학병원 연구진의 협력을 통해 24주간 다면적 노쇠 예방 중재를 제공한 결과, 참가 노인의 ‘장애 없는 생존 기간’이 6.5개월 연장되고 1인당 약 1,000만 원에 달하는 의료‧요양 비용 절감 효과(비용 대비 편익 약 8.8배)를 확인하였다[14,15]. 경남 남해군은 질병관리청의 ‘소지역 건강격차 해소사업’의 일환으로 건강 수준이 낮고, 만성질환자가 많은 면(面) 단위 지역 주민을 대상으로 노쇠예방 운동교실, 단백질 보충, 방문건강관리를 통합 제공함으로써 대상자의 신체기능과 영양지수를 개선하였다[16]. 또한 부산광역시는 동(洞) 단위 마을건강센터와 주민 참여형 건강파트너 인력을 활용하여 지역 밀착형 노쇠 예방 모델을 활발히 운영 중이다[17].

### 3. 지역사회 중심 노쇠 예방 사업의 한계

여러 지자체의 노쇠 예방 사업을 통해 노쇠의 가역적 회복 가능성과 지역사회 복합 중재의 효과가 검증되었음에도 불구하고, 많은 보건소는 노쇠 예방 사업을 추진하는 데 다음과 같은 구조적 어려움을 겪고 있다.

#### 1) 노쇠의 선제적 발굴 및 예방적 개입의 한계

여러 보건의료 전문가들이 제기하는 근본적인 문제 중 하나는 현행 지역사회 보건 체계가 치료 및 돌봄 중심의 사후 대응에 비중을 두고 있다는 점이다. 현재 보건소의 노쇠 관리는 취약계층 대상의 방문건강관리나 일반 건강증진 사업 중심으로 분절되어 운영되어 왔으며, 기능 저하가 시작되는 초기 대상자를 조기에 포착하여 예방 사업으로 연계하는 경로가 제한적이다[18-20]. 또한, 보건소의 기존 방문건강관리 전담 인력은 이미 취약계층 관리만으로도 업무 부담이 크기 때문에 신규 노쇠 예방 사업 대상자 전체에게 선제적인 중재 서비스를 제공하기에는 현실적인 제약이 따른다[21]. 이로 인해 노쇠 관리의 핵심이 전노쇠 및 초기 노쇠 단계에서의 신속한 개입으로 기능 회복을 도모하는 것임에도 불구하고, 실제 현장에서는 건강 취약성이 심화되거나 중증 노쇠로 진행된 이후에야 관리가 시작되는 노쇠 관리의 한계가 반복되고 있다.

#### 2) 표준화 매뉴얼 부재 및 사업 지속 가능성의 한계

두 번째는 현장 실무자가 활용할 수 있는 표준화된 사업 매뉴얼 부재와 사업 담당자의 기획‧운영 역량 한계이다. 보건소 보건사업 담당자 대상 조사에 따르면 노쇠 예방 사업 추진 시 가장 큰 애로사항으로 ‘프로그램 기획 및 운영 역량 부족’을 꼽았으며, 응답자의 25.4%가 최우선 지원 요구사항으로 ‘표준화된 사업 지침 제공’을 선택하였다[22]. 그간 지자체에서 거둔 일부 성공 사례 또한 지자체장 및 보건소장의 강력한 의지, 담당자의 개인적 역량, 또는 외부 전문가(대학 연구진 등)의 밀착지원에 의존한 측면이 크다. 이로 인해 담당자 인사이동이나 외부 지원 종료 시 사업이 지속되지 못하는 한계를 보였다. 이는 현장 실무자가 즉시 활용할 수 있는 표준화된 선별‧평가 도구, 근거 기반 중재 프로그램, 성과 평가 가이드라인 등 국가 차원의 표준 매뉴얼 제공이 시급함을 보여준다.

#### 3) 근거 기반 기획을 위한 지역 단위 노쇠 데이터 부재

마지막으로, 근거 기반 기획을 위한 지자체 단위 노쇠 현황 데이터의 부재이다. 그간 노쇠 유병률은 「노인실태조사」, 「국민건강영양조사」 등 국가 단위 조사나 일부 연구결과를 활용해 왔다. 따라서 지자체(보건소) 현장에서는 지역 내 노쇠‧전노쇠 유병률과 관련된 인구사회학적 요인을 연계 분석할 수 있는 지자체 단위 통계가 없어, 지역의 노쇠 실태를 객관적으로 진단하고 근거 기반 맞춤형 사업을 기획하는 데 근본적인 어려움을 겪어왔다. 노쇠는 성별, 연령, 거주 환경, 가구 형태 등에 따라 유병 양상이 다르게 나타나므로, 사업의 실효성을 극대화하기 위해서는 지자체별 통계 자산에 기반한 맞춤형 전략 수립이 필수적으로 전제되어야 한다.

## 결 론

노쇠는 전노쇠 및 초기 노쇠 단계에서 선제적으로 발견하고 맞춤형 중재를 제공할 경우 정상 상태로 회복이 가능한 ‘가역적 역동성’을 지닌다. 그러나 현재 보건소 사업은 취약계층 중심의 방문건강관리나 통합건강증진사업과 같은 보편적인 건강관리 서비스에 치중되어 있어, 일반 노인 및 초기 위험군으로의 대상 확장에 한계를 보이고 있다. 또한 표준화된 노쇠 사업 매뉴얼과 지역 맞춤형 데이터의 부재로 선제적인 노쇠 예방 체계를 구축하는 데 어려움을 겪고 있다. 보건소 담당자 설문에서도 확인된 바와 같이, ‘표준화된 사업 지침 제공’이 최우선 과제로 나타난 점은 전국 보건소 현장에서 즉시 활용 가능한 표준화된 가이드라인 마련이 매우 시급함을 명확히 시사한다.

다행히 최근 질병관리청을 중심으로 이러한 현장의 한계를 극복하기 위한 정책적 기반 조성이 적극적으로 추진되고 있다. 그간 우리나라에 통일된 국가 노쇠 예방 사업 기준이 부재했던 상황을 개선하고자, 노쇠에 대한 대국민 인식 개선을 위한 ‘국가 노쇠 정의 표준안’과 ‘노쇠 인식개선 메시지’를 개발하고 있으며, 보건소 현장에 즉시 적용 가능한 ‘표준 사업 매뉴얼’ 마련에도 앞장서고 있다[23]. 아울러 2026년 지역사회건강조사에 ‘한국형 노쇠선별도구(K-FRAIL)’ 지표를 신규 도입함으로써, 전국 지자체 단위의 객관적인 노쇠 현황 통계를 산출할 수 있는 제도적 기반을 마련하였다[24].

이러한 정책적 전환점을 맞아, 지자체는 향후 산출되는 지역사회건강조사 데이터를 바탕으로 지역 노인의 성별, 연령별, 거주지별, 가구 형태별 특성을 정밀하게 연계 분석하고 이를 사업 기획에 적극 반영해야 한다. 노쇠는 인구사회학적 특성에 따라 위험도와 개입 전략이 달라지는 만큼, 중앙정부는 최소한의 서비스 품질을 보장하는 표준 가이드라인을 제공하되, 각 지자체가 인구구조와 지역 특성에 맞게 우선순위를 설정하고 맞춤형 프로그램을 자율적으로 구성할 수 있도록 지자체의 기획‧운영 역량을 강화해야 할 것이다.

이와 함께 효과적인 노쇠 예방을 위해서는 지역사회 전반에 노쇠가 자연스러운 노화 현상이 아니라 예방과 관리로 회복 가능한 상태임을 인지하는 분위기가 조성되어야 한다. 경로당, 복지관, 마을회관 등 어르신들의 일상적 생활 접점을 중심으로 노쇠 자가 진단 및 예방 수칙을 적극 홍보하고, 지역 주민이 스스로 건강을 살피는 건강 친화적 분위기를 구축할 때 선제적 대상자 발굴과 주민 자발적 참여가 촉진될 수 있다.

더불어 보건소의 한정된 전담 인력만으로는 급증하는 노쇠 위험군 전체를 직접 관리하는 데에 한계가 있으므로, 해외의 ‘프레일 서포터(일본)’나 지자체의 ‘주민 건강파트너(부산)’ 사례처럼 주민 봉사자, 노인 일자리 인력, 지역 보건‧의료기관 및 복지시설 등 지역사회의 다양한 인적‧물적 자원을 유기적으로 연결하고 조직화하여 지역사회 전체가 함께 작동하는 민관 협력 생태계로의 기능 전환을 도모해야 한다.

특히 국내 사례에서도 볼 수 있듯이 중재 프로그램 종료 후에도 어르신들의 회복된 신체 기능과 건강 행동이 유지될 수 있도록 ‘지속 가능한 사후관리 체계(자조 모임 등)’를 마련하는 것 역시 중요한 과제이다. 프로그램 수료자를 지역 내 자조 모임, 경로당 동아리, 주민 운동교실 등과 연계하고, 주민 리더나 건강파트너를 활용해 일상 속 운동‧영양 관리가 끊김없이 이어지는 선순환 유지 체계를 구축하여 중재 효과의 장기적 지속성을 담보해야 한다.

결국 국가 차원의 표준화된 매뉴얼 및 인식 개선 기반 위에 지역 통계에 근거한 지자체 맞춤형 기획 역량과 지역 인적자원을 동원하는 민관 협력 체계가 긴밀히 결합할 때 비로소 초고령사회에 대응하는 실효성 있고 지속 가능한 지역 맞춤형 노쇠 예방 체계가 완성될 것이다.

## Figures and Tables

**표 1:** 노인의 노쇠 관련 주요 현황 및 위험 요인

구분		주요 현황	예상되는 위험 요인
성별	여성	- 65세 이상 노쇠 5.9%a), 65–69세 근감소 5.8%b) (남성 대비 2배↑) - 80세 이상 악력 저하율 41.5%b)	기초 근육량 부족, 호르몬 변화, 사회경제적 취약성으로 인한 조기 발현 및 생애 후반 장기 지속
	남성	- 65세 이상 노쇠 2.8%a), 65-69세 근감소 2.6%b) - 70세 이후 근감소 위험 급증b)	70세 이후 급격한 근손실 및 만성질환 누적으로 노쇠 유병 위험의 급격한 증가
연령별	전기 고령 (65–74세)	- 악력 저하군 우울장애 14.3%b) (정상군 2.0% 대비 7배↑)	근력 약화, 은퇴 등 사회적 역할 상실이 정신건강(우울) 위험 증가와 밀접하게 연관
	후기 고령 (75세 이상)	- 악력 저하군 걷기 미실천 64.4%b) (정상군 54.1%) - 저작 불편 호소율 42.0%b) (정상군 32.6%)	신체활동 감소+구강 기능 저하+영양 부실 → 신체‧영양 악순환
환경 및 가구 특성	농어촌	- 노쇠 6.0%, 전노쇠 36.1%a) (도시: 4.1%/30.9%)	높은 평균 연령, 의료‧운동 인프라 접근성 한계, 사회적 교류 단절
	독거 가구	- 노쇠 7.1%a) (노인부부 2.8% 대비 2.5배↑)	부실한 영양 섭취, 정서적 적막감, 신체활동 감소 → 우울/인지 기능 저하 가속

^a)^Data from Kang et al. (2023 National Survey of Older Koreans; 2023) [5]. ^b)^Data from the Korea Disease Control and Prevention Agency (https://www.kdca.go.kr/bbs/kdca/42/312118/artclView.do) [9].
